# Validation of [^125^I]CPCR4.3 as an investigative tool for the sensitive and specific detection of hCXCR4 and mCXCR4 expression in vitro and in vivo

**DOI:** 10.1186/s13550-019-0545-2

**Published:** 2019-08-13

**Authors:** Margret Schottelius, Marina Ludescher, Frauke Richter, Tobias G. Kapp, Horst Kessler, Hans-Jürgen Wester

**Affiliations:** 1 Chair for Pharmaceutical Radiochemistry, Technische Universität München, Walther-Meissner-Strasse 3, 85748 Garching, Germany; 2 Chemistry Department, Institute for Advanced Study, Technische Universität München, Lichtenbergstr. 4, 85747 Garching, Germany

**Keywords:** CXCR4, Chemokine receptor, Cancer, Radioligand, CPCR4.3, Receptor expression, Species selectivity

## Abstract

**Background:**

The development and clinical translation of [^68^Ga] Pentixafor has substantially promoted the relevance of non-invasive PET imaging of CXCR4 expression in a broad spectrum of diseases, including cancer and inflammation. Its pronounced selectivity for the human receptor (hCXCR4), however, precludes the use of [^68^Ga] Pentixafor for imaging receptor expression and dynamics in CXCR4-related diseases in endogenous mouse models. To overcome this restriction, [^125^I]CPCR4.3, a structurally related pentapeptide ligand, has been evaluated as a preclinical tool for efficient in vitro and in vivo targeting of hCXCR4 and mCXCR4.

**Results:**

Compared to the reference [^68^Ga] Pentixafor, [^125^I]CPCR4.3 showed 2.4- to 11-fold increased specific binding to human cancer cell lines with different hCXCR4 expression levels (Jurkat, Daudi, HT-29, SH-5YSY, MCF-7, LNCaP) as well as strong and highly specific binding to mCXCR4 expressing cells (mCXCR4-transfected CHO cells, Eμ-myc 1080, 4 T1), which was not detectable for [^68^Ga]Pentixafor. This is the consequence of the equally high affinity of iodo-CPCR4 to hCXCR4 and mCXCR4 (IC_50_ = 5.4 ± 1.5 and 4.9 ± 1.7 nM, respectively) as opposed to [^nat^Ga] Pentixafor (hCXCR4: 42.4 ± 11.6 nM, mCXCR4: > 1000 nM). Additionally, [^125^I]CPCR4.3 showed enhanced tracer internalization (factor of 1.5–2 compared to the reference). In vivo biodistribution studies in immunocompetent Black Six and immunocompromised CD-1 nude mice showed predominant hepatobiliary excretion of [^125^I]CPCR4.3 (logP = 0.51), leading to high activity levels in liver and intestines. However, [^125^I]CPCR4.3 also showed high and specific accumulation in organs with endogenous mCXCR4 expression (spleen, lung, adrenals), even at low receptor expression levels.

**Conclusions:**

Due to its excellent hCXCR4 and mCXCR4 targeting efficiency, both in vitro and in vivo, [^125^I]CPCR4.3 represents a sensitive and reliable tool for the species-independent quantification of CXCR4 expression. Its suboptimal clearance properties will certainly restrict its use for in vivo imaging applications using ^123^I (for SPECT) or ^124^I (for PET), but due to its high and specific accumulation in mCXCR4 expressing tissues, [^125^I]CPCR4.3 holds promise as a powerful preclinical tool for the investigation and quantification of CXCR4 involvement and kinetics in various murine disease models via, e.g., biodistribution and autoradiography studies.

## Introduction

**Fig. 1:**
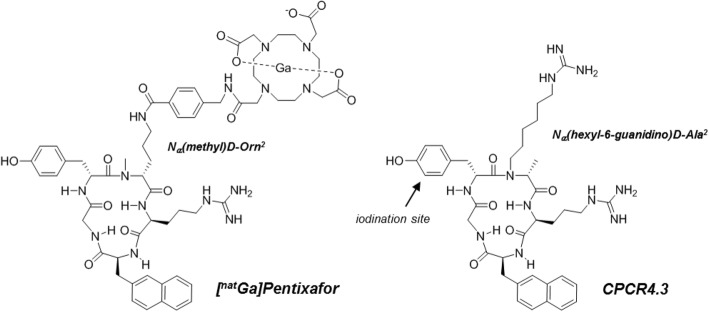
Structures of [^nat^Ga] Pentixafor and CPCR4.3; please note the central structural modification introduced into the peptide backbone, i.e. the shift of the functionalized alkyl chain from C_α_ of d-Orn^2^ (in Ga-Pentixafor) to N_α_ of d-Ala^2^ in CPCR4.3

In recent years, non-invasive in vivo imaging of chemokine receptor 4 (CXCR4) expression has gained increasing clinical interest, based on the important role of CXCR4 expression in cancer development, progression, and metastasis and its pivotal involvement in inflammatory conditions, and driven by the development and clinical translation of [^68^Ga] Pentixafor (Fig. 1) [1, 2]. In contrast to other CXCR4-targeted radiotracers evaluated in humans, e.g., [^64^Cu]AMD3100 [3] or the 14-amino acid peptide [^68^Ga]NOTA-NFB [4], the cyclic pentapeptide [^68^Ga] Pentixafor shows rapid renal excretion and very low non-specific background accumulation, alongside with high affinity and selectivity for human CXCR4. These features have led to widespread clinical imaging applications, ranging from imaging of various hematological and solid cancers to different inflammatory conditions (for a comprehensive review see [5]).

Although it has proven to be a powerful and sensitive tool for quantitative PET imaging of CXCR4 expression in humans, the applicability of [^68^Ga] Pentixafor for preclinical investigations of CXCR4-related pathologies in mouse models is substantially restricted by its pronounced selectivity for the human CXCR4 receptor (hCXCR4) versus the murine CXCR4 homolog (mCXCR4). To date, there is only one report on the use of [^68^Ga] Pentixafor for imaging of inflammation after myocardial infarction in a mouse model [6], and although some CXCR4-specific signal was observed, signal-to-background ratios were very low.

Thus, to allow more sensitive imaging of endogenous mCXCR4 expression in mice as well as an accurate assessment of CXCR4-related side effects (off-target effects) in CXCR4-targeted therapeutic interventions such as CXCR4-directed endoradiotherapy [7], high-affinity tracers for mCXCR4 are indispensable. Optimally, such tracers should have equal affinity for mCXCR4 and hCXCR4 to ensure clinical translatability.

As shown in the literature, radiolabeled AMD3465 analogues do show affinity to mCXCR4, as indicated by a certain extent of blockable ligand uptake in mCXCR4-expressing tissues such as spleen or bone marrow [8]. The same has been observed for T-140 analogs, e.g., [^18^F]FBz-TN14003 [9]. However, mCXCR4 affinity has not been determined for these compounds.

In a different context, we recently reported on the identification of a conformationally restricted FC-131-derived cyclopentapeptide with subnanomolar affinity for hCXCR4 [10]. Surprisingly, this peptide (cyclo(-d-Tyr^1^-d-[*N*-hexyl-6-guanidino]Ala^2^-Arg^3^-Nal^4^-Gly^5^-), CPCR4.3, Fig. 1) also showed very high mCXCR4 affinity in a preliminary screening. Unfortunately, the compact and highly optimized pentapeptide structure does not allow for substitution of side chain functionalities without compromising receptor affinity, and their functionalization with, e.g., chelators for radiometal labeling is chemically complex and challenging. Thus, to generally evaluate the suitability of the CPCR4.3 scaffold as a high-affinity tool for the species-independent detection and quantification of CXCR4 expression in vivo, we selected the corresponding radioiodinated analog [^125^I]CPCR4.3 as the lead compound for this proof-of-concept study.

The hCXCR4- and mCXCR4-specific binding of [^125^I]CPCR4.3 was investigated in a panel of human and murine cancer cell lines with different levels of endogenous CXCR4 expression as well as CHO-K1 cells transiently transfected with hCXCR4 and mCXCR4 (positive controls) and compared to the hCXCR4 selective reference compound [^68^Ga]Pentixafor. To evaluate the in vivo mCXCR4 targeting efficiency of [^125^I]CPCR4.3, comparative biodistribution studies were performed in non-tumor-bearing Black Six (immunocompetent) and CD-1 nude (immunocompromised) mice.

## Materials and methods

### Synthesis and radiolabeling

CPCR4.3 was synthesized as described [10]. The corresponding 3-iodo-Tyr-analog was obtained by iodination of CPCR4.3 with N-iodo-succinimide [11].

The reference ligand [^68^Ga] Pentixafor was prepared according to a previously established protocol [2]. Radioiodination of CXCR4.3 was carried out using the IodoGen® method. Briefly, 100–200 μg of peptide were dissolved in 0.5 mL TRIS iodination buffer (25 mM Tris·HCl, 0.4 M NaCl, pH 7.5) and transferred to an Eppendorf reaction tube coated with 150 μg of IodoGen®. Upon addition of [^125^I] NaI (18–20 MBq, Hartmann Analytik, Braunschweig, Germany), the reaction vessel was briefly vortexed and the labeling reaction was allowed to proceed for 15 min at RT. The peptide solution was then removed from the insoluble oxidizing agent. Separation of [^125^I]CPCR4.3 from unlabeled precursor was achieved using gradient RP-HPLC (column: Nucleosil 100 C18 (5 μm, 125 × 4.0 mm; CS GmbH, Langerwehe, Germany), gradient: 22–42% ethanol (0.5% acetic acid) in water (0.5% acetic acid) within 20 min, flow: 1 mL/min).

For in vitro binding and uptake studies, the HPLC product fraction was used as such and diluted to the required concentration using the respective assay medium. For biodistribution experiments, excess ethanol was removed by bubbling an argon stream through the product fraction at 90 °C for 20 min. [^125^I]CPCR4.3 was then reconstituted to an activity concentration of app. 1 MBq/100 μL using PBS and was then used for the in vivo study.

### Lipophilicity

The lipophilicity (log P) of [^125^I]CPCR4.3 was determined via a modified shake-flask method as described previously [12].

### Cell culture

Unless stated otherwise, the cell lines used in this study were purchased from ECACC (European Collection of Authenticated Cell Cultures, Salisbury, UK), DSMZ (Deutsche Sammlung von Mikroorganismen und Zellkulturen, Braunschweig, Germany) or ATCC (American Type Culture Collection, Wesel, Germany). Jurkat human T lymphocyte cells (DSMZ: ACC282), SH-SY5Y human neuroblastoma cells (ECACC: 94030304), and 4 T1 mouse mammary carcinoma cells (ATCC: CRL-2539) were maintained in RPMI 1640 medium supplemented with 10% fetal calf serum (FCS). Daudi human Burkitt lymphoma cells (ECACC: 85011437) were cultured in RPMI-1640 medium supplemented with 10% FCS, 2 mM l-glutamine, 1% non-essential amino acids, 50 μM β-mercaptoethanol, and 100 units/mL of penicillin/streptomycin (P/S). Eμ-myc 1080 mouse B cell lymphoma cells (kindly provided by Prof. Ulrich Keller, Charité, Berlin) [13] were grown in RPMI 1640 medium supplemented with 20% FCS, 1% NEA, 100 units/mL of P/S, and 0.1% 2-mercaptoethanol. The human colon carcinoma cell line HT-29 (ECACC: 91072201), the breast cancer cell line MCF-7 (ECACC: 86012803), and LNCaP human prostate carcinoma cells (DSMZ: ACC256) were cultured in DMEM/F-12 medium with Glutamax-I (1:1) supplemented with 10% FCS. CHO-K1 cells (Chinese hamster ovary cells, ECACC: 85051005) were cultured in RPMI-1640 medium supplemented with 10% FBS, 2 mM l-glutamine, and 100 units/mL of P/S. All cell lines were maintained at 37 °C in a humidified 5% CO_2_ atmosphere. Media and supplements were obtained from Biochrom (Berlin, Germany) or Gibco (life technologies, Darmstadt, Germany).

In the assay medium used for internalization studies, FCS was replaced by 5% bovine serum albumin (BSA; Sigma, St. Louis, USA). For cell counting, a Countesse automated cell counter (Invitrogen, Carlsbad, USA) was used.

### Comparative binding studies using various cell lines

CXCR4-mediated cellular uptake of [^125^I]CPCR4.3 and the reference [^68^Ga] Pentixafor was investigated in various cell lines with different endogenous hCXCR4 (Jurkat, Daudi, HT-19, MCF-7, SH-5YSY, LNCaP) and mCXCR4 (Eμ-myc 1080, 4 T1) expression levels. For data validation, CHO-K1 cells transiently transfected with hCXCR4 and mCXCR4 [2] were also included, with non-transfected CHO-K1 cells serving as CXCR4 negative controls.

In the case of suspension cells (Daudi, Jurkat, Eμ-myc 1080), samples containing 2 × 10^5^ cells in assay medium were incubated with [^125^I]CPCR4.3 (0.1 nM) or the reference [^68^Ga] Pentixafor (1 nM) at 37 °C for 60 min in the presence (non-specific binding) or absence (control) of 100 μM AMD3100 (*n* = 3 per concentration, total sample volume: 250 μL). After incubation, the tubes were centrifuged (3 min, 1300 rcf, Megafuge 1.0, Heraeus Thermo Scientific) and the supernatant was carefully removed. After washing twice with 200 μL of cold HBSS, the amount of bound radioligand in the cell pellet was quantified using a γ-counter (WALLAC; 1480 WIZARD™ 3″).

In the case of the adherent cell lines (CHO, 4 T1, HT-19, MCF-7, SH-5YSY, LNCaP), app. 150,000 cells/well were seeded into PLL-coated 24-well plates (Greiner, Bio One, Frickenhausen, Germany) on the day prior to the experiment. On the day of the experiment, the culture medium was removed, and the cells were left to equilibrate in 200 μL of assay medium (RPMI + 5% BSA) at 37 °C for a minimum of 15 min before the experiment. Then, the cells were incubated with [^125^I]CPCR4.3 (0.1 nM) or the reference [^68^Ga] Pentixafor (1 nM) at 37 °C for 60 min in the presence (non-specific binding) or absence (control) of 100 μM AMD3100 (*n* = 3 per concentration, total sample volume: 250 μL). Upon incubation, the incubation medium was removed, and cells were rinsed twice with 250 μL of HBSS and lysed using 300 μL of 1 N NaOH. The lysate was transferred to vials and combined with 250 μL of HBSS used for rinsing the wells. Quantification of the amount of free and bound activity was performed in an Automatic Gamma Counter.

### Determination of IC_50_

Affinities for human and murine CXCR4 (hCXCR4 and mCXCR4) were determined in competitive binding assays (IC_50_) [14] using either Jurkat or Eμ-Myc 1080 mouse lymphoma cells (2 × 10^5^ cells/sample) in Hank’s buffered salt solution (1% BSA) and [^125^I]CPCR4.3 as radioligand. To allow data normalization, [^nat^Ga] Pentixafor and FC-131 were included as references in this study. Experiments were performed in triplicate with *n* = 3 per concentration in each experiment. IC_50_ values were calculated using GraphPad Prism 6.01 (Graph Pad Software, San Diego, USA).

### Internalization and externalization

The internalization of [^125^I]CPCR4.3 and [^68^Ga] Pentixafor into the human cancer cell lines HT-29, SH-5YSY, MCF-7, and LNCaP was investigated in analogy to a previously published protocol [15]. Non-specific internalization was determined in the presence of 100 μM AMD3100.

To determine ligand washout and recycling kinetics, cells (CHO-hCXCR4, CHO-mCXCR4, as well as HT-29 and MCF-7 cells as representative cell lines endogenously expressing hCXCR4) were first incubated with [^125^I]CPCR4.3 (0.1 nM) at 37 °C for 30 min and washed with HBSS. In the experiment allowing ligand recycling, 250 μL of assay medium were added to the wells (*n* = 3). In the experiment inhibiting ligand recycling, 250 μL of assay medium containing 100 μM AMD3100 were added to the wells (*n* = 3). Subsequently, cells were incubated at 37 °C for 5, 15, 30, and 60 min, respectively. The supernatant was removed and combined with 250 μL of HBSS used for rinsing the cells. This fraction represents the amount of externalized ligand at the respective time point. The following lysis of the cells was performed as described for the radioligand binding study.

### In vivo biodistribution studies

Animal experiments were performed in accordance with the current animal welfare regulations in Germany (approval #55.2-1-54-2532-71-13).

To investigate the biodistribution of [^125^I]CPCR4.3, approximately 550 kBq (15 μCi) of [^125^I]CPCR4.3 in a total volume of 100 μL of PBS (pH 7.4) were injected intravenously (i.v.) into the tail vein of immunocompetent Black Six mice (*n* = 4) under isoflurane anesthesia. To determine mCXCR4 specificity of ligand accumulation, 50 μg AMD3100/mouse were coinjected (*n* = 4). The animals were sacrificed 60 min post injection (p.i.), and the organs of interest were dissected. The radioactivity was measured in weighted tissue samples using a γ-counter. Data are expressed in % ID/g tissue (mean ± SD). For a comparative biodistribution study in an immunodeficient mouse strain, CD-1 nu/nu mice were used. Statistical analysis (one-tailed *t* test) of the separate biodistribution data sets was performed using Microsoft Excel.

## Results and discussion

### CXCR4-specific cellular uptake

**Fig. 2:**
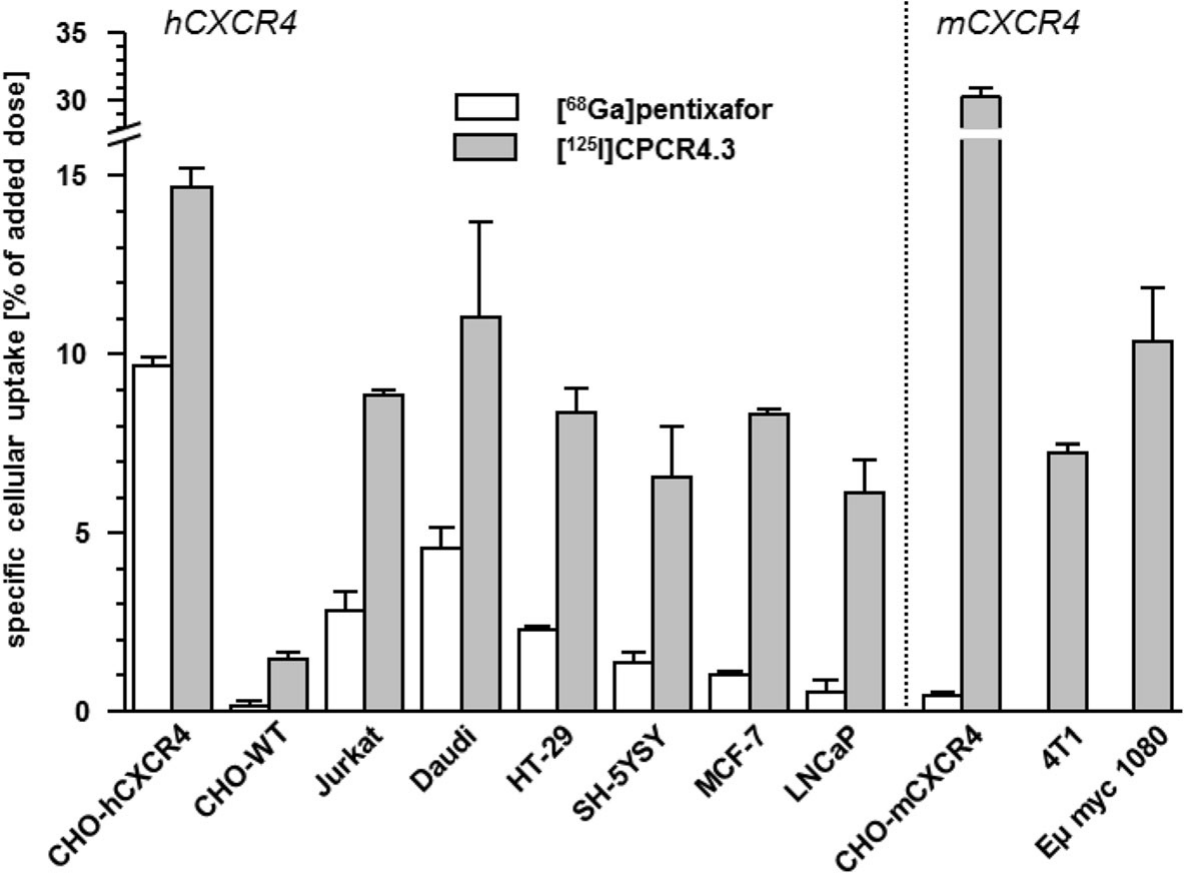
CXCR4-specific cellular uptake of [^125^I]CPCR4.3 (0.1 nM) and [^68^Ga] Pentixafor (1 nM) in different hCXCR4- and mCXCR4 expressing cell lines (60 min, 37 °C). CHO-WT (non-transfected CHO cells) were included as negative control. Data are corrected for non-specific binding in the presence of 100 μM AMD3100. Experiments were performed in triplicate, and data are means ± SD

Figure 2 summarizes the specific binding (total cellular binding corrected by non-specific binding in the presence of 100 μM AMD3100) of [^125^I]CPCR4.3 and the reference ligand [^68^Ga] Pentixafor to a panel of human and murine cancer cell lines with different levels of endogenous CXCR4 expression as well as to CHO-K1 cells transiently transfected with h- and mCXCR4 (positive controls) after a 60-min incubation (37 °C).

As shown, hCXCR4-specific binding of both [^125^I]CPCR4.3 and [^68^Ga] Pentixafor follows the same pattern; both ligands exhibit highest uptake in the hCXCR4-transfected control cell line CHO-hCXCR4 and low binding to the negative control cells CHO-WT (9.8 and 1.3% of positive control, respectively). For [^125^I]CPCR4.3, the level of non-specific binding was generally higher than for [^68^Ga]Pentixafor: in the presence of 100 μM AMD3100, 3.7 ± 0.6% of the added [^125^I]CPCR4.3 activity was found to be non-specifically bound to the cells after 60 min, independently from the cell line used, with only 1.3 ± 0.1% of the [^68^Ga] Pentixafor activity being non-specifically bound under the same conditions. This is most probably the result of the increased lipophilicity of [^125^I]CPCR4.3 (log *P* = 0.51 vs log *P* = − 2.9 for [^68^Ga]Pentixafor), favoring non-specific interactions with the cell membrane.

In the cell lines with endogenous hCXCR4 expression, uptake levels of both [^125^I]CPCR4.3 and the reference [^68^Ga] Pentixafor follow the same pattern, with the human lymphoma cell lines Jurkat and Daudi showing the highest uptake. These findings correlate well with the documented high (Daudi, Jurkat) [2]; moderate (HT-29 [8], SH-5YSY [16]); and low (MCF-7 [17], LNCaP [18]) CXCR4 expression in the respective cell lines. Generally, for all hCXCR4-expressing cell lines, specific binding of [^125^I]CPCR4.3 was found to be 2.4- (Daudi cells) to 11-fold (LNCaP cells) enhanced compared to [^68^Ga] Pentixafor, indicative of a higher affinity of the radioiodinated ligand to hCXCR4 and consequently a more sensitive detection of low-level CXCR4 expression.

Interestingly, while [^68^Ga] Pentixafor shows virtually no binding to mCXCR4 [2], [^125^I]CPCR4.3 binds strongly and specifically to murine CXCR4, both in the mCXCR4-transfected CHO control cells and the murine lymphoma (Eμ-myc 1080, high CXCR4 expression) and breast cancer (4 T1, moderate CXCR4 expression [19]) cell lines, respectively.

### CXCR4 affinity

Based on the initial ligand binding experiment demonstrating the species independence of cellular [^125^I]CPCR4.3 binding, radioiodinated CPCR4.3 was used as the “universal” radioligand in the subsequent determination of the hCXCR4 and mCXCR4 affinity of CPCR4.3 and its 3-iodo-Tyr- counterpart (iodo-CPCR4.3) in comparison to the commonly used reference ligand FC-131 and [^nat^Ga]pentixafor.

As shown in Table 1, [^nat^Ga] Pentixafor shows the already documented pronounced selectivity for hCXCR4 over mCXCR4 [2]. The other reference ligand, FC-131, shows a less pronounced species selectivity and more than 3-fold higher hCXCR4 affinity than [^nat^Ga] Pentixafor under the assay conditions used in this study.

**Table 1:** Binding affinities (IC_50_ in nM) of the reference ligands FC-131 and Ga-pentixafor as well as various CPCR4.3 and iodo-CPCR4.3 to human and mouse CXCR4 (hCXCR4, mCXCR4). Affinities were determined using Jurkat human T cell leukemia and Eμ-Myc1080 mouse B cell lymphoma cells, respectively (200,000 cells/sample), and [^125^I]CPCR4.3 as the radioligand. Each experiment was performed in triplicate, and results are means ± SD from three separate experiments

Peptide	IC_50_ [nM] to *h*CXCR4	IC_50_ [nM] to *m*CXCR4
FC-131	12.4 ± 3.1	119 ± 69
Ga-Pentixafor	42.4 ± 11.6	> 1000
CPCR4.3	2.8 ± 1.1	0.8 ± 0.1
iodo-CPCR4.3	5.4 ± 1.5	4.9 ± 1.7

Compared to the two reference ligands, CPCR4.3 shows substantially improved hCXCR4 affinity. This explains both the improved ligand binding of [^125^I]CPCR4.3 to hCXCR4 expressing cells in comparison to [^68^Ga] Pentixafor observed in the initial radioligand binding study (Fig. 2) and also the resulting improved “detectability” of CXCR4 expression, even at low levels, by [^125^I]CPCR4.3. Please note, however, that due to altered assay conditions (different cell number, different radioligand), the numerical values for the affinity of CPCR4.3 and [^nat^Ga] Pentixafor differ from previously reported data [10].

Most notably, CPCR4.3 shows outstandingly high affinity toward mCXCR4. Iodination of Tyr^3^ in CPCR4.3 leads to a slight loss in hCXCR4 affinity, whereas mCXCR4 affinity is app. 6-fold decreased compared to the native peptide ligand. Nevertheless, iodo-CPCR4.3 displays very high and identical affinity both to the human and the murine CXCR4 receptor and thus—as the first pentapeptide antagonist in the FC-131 family—overcomes the limiting species selectivity.

As shown in our previous study on the parent peptide CPCR4.3, the successive implementation of small structural changes in the pentixafor backbone, i.e., introduction of the so-called “peptoid structure” [10], in which the side chain of Orn^2^ is shifted to the neighboring N_α_ nitrogen, and finetuning of the length of the alkyl chain (C_3_ in D-Arg^2^ to C_6_ in CPCR4.3), led to a 75-fold increase in hCXCR4 affinity. This was attributed to the conformational rigidity of CPCR4.3, allowing it to make contact with a receptor region that was previously unexplored by the parent peptides [10, 20]. Given the limited homology of only 88.86% between hCXCR4 and mCXCR4 (source: alignment search in https://www.uniprot.org) and the complex interaction of CXCR4-ligands with several transmembrane helices and extracellular loops of the respective CXCR4 proteins [21], alongside with the documented probability of alternative binding modes, it was both an unexpected finding and a positive surprise that [^125^I]CPCR4.3 is able to target both receptors with the same affinity.

### Internalization and externalization

**Fig. 3:**
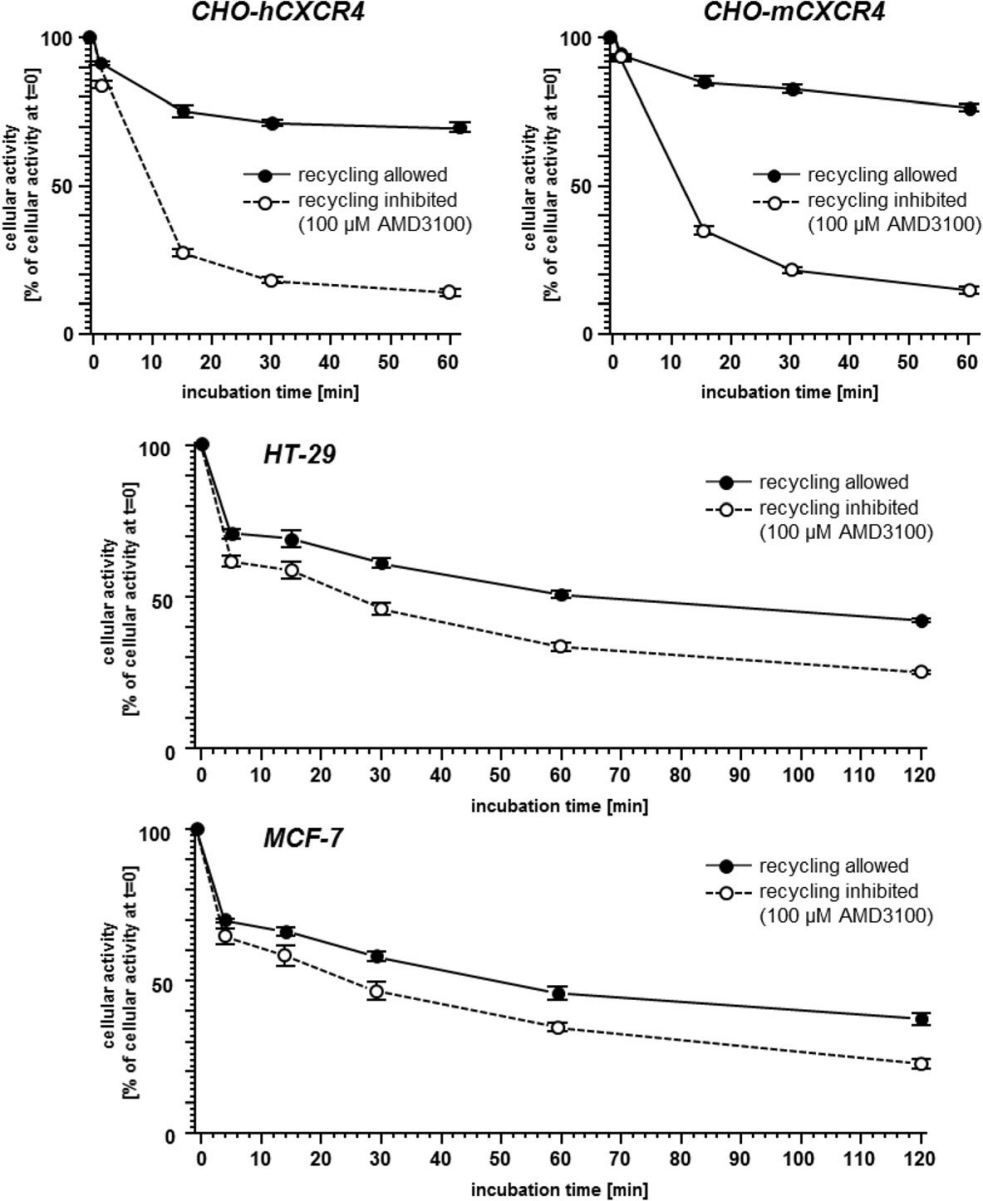
Exemplary eternalization kinetics of [^125^I]CPCR4.3 in different hCXCR4 (CHO-hCXCR4, HT-29, MCF-7) and mCXCR4 (CHO-mCXCR4) expressing cell lines. Externalization kinetics at 37 °C were determined under conditions allowing (assay medium only) or inhibiting (100 μM AMD3100 in assay medium) ligand re-binding/recycling. Data are given as total cellular activity (% of cellular activity at *t* = 0). Each experiment was performed in triplicate, and results are means ± SD

To further characterize [^125^I]CPCR4.3, its internalization and externalization properties (Table 2 and Fig. 3, respectively) were investigated in different adherent cell lines and compared to [^68^Ga]Pentixafor. As summarized in Table 2, the internalization of [^125^I]CPCR4.3 into the hCXCR4 expressing cell lines investigated was found to be twice as efficient as that of the reference—with the sole exception of the HT-29 colon carcinoma cells, where comparable values were obtained for [^125^I]CPCR4.3 and [^68^Ga]Pentixafor.

**Table 2:** Internalization efficiency (internalized activity in % of total cellular activity) of [^125^I]CPCR4.3 (0.1 nM) and the reference [^68^Ga] Pentixafor (1 nM) in different human cancer cell lines after 60 min at 37 °C. Data are corrected for non-specific binding/internalization in the presence of 100 μM AMD3100. Each experiment was performed in triplicate, and results are means ± SD

Cell line	[^125^I]CPCR4.3 [% of total]	[^68^Ga] Pentixafor [% of total]
HT-29	45.7 ± 5.5	48.2 ± 7.7
SH-5YSY	65.6 ± 21.9	32.0 ± 6.7
MCF-7	68.4 ± 3.3	29.4 ± 5.7
LNCaP	54.0 ± 15.6	32.2 ± 25.1

To evaluate the extent of tracer washout and ligand recycling as well as eventual differences in ligand processing between hCXCR4 and mCXCR4 expressing cells, externalization and recycling kinetics of [^125^I]CPCR4.3 were investigated in CHO-K1 cells transiently transfected with hCXCR4/mCXCR4. Figure 3 shows exemplary tracer release kinetics. As anticipated from the internalization data in Table 2, [^125^I]CPCR4.3 shows rapid reuptake into hCXCR4 expressing CHO cells, when reinternalization is allowed, leading to the apparent retention of 69.6 ± 0.1% of the initial cellular activity after 60 min. In contrast, when reinternalization is prohibited by an excess of unlabeled competitor in the external medium, only 14.0 ± 0.3% of the initial cellular activity are retained. Nearly identical values were observed for [^125^I]CPCR4.3 using mCXCR4 expressing cells (retention of 76.7 ± 0.7% and 14.7 ± 0.4% of the initial cellular activity under the respective assay conditions), demonstrating species independent externalization and recycling kinetics for [^125^I]CPCR4.3.

In the two representative cell lines with endogenous hCXCR4 expression (HT-29 and MCF-7), apparent cellular retention of [^125^I]CPCR4.3 under conditions allowing ligand recycling is reduced compared to the transfected cell lines (with only 50.8 ± 1.2% (HT-29) and 45.2 ± 1.9% (MCF-7) of the initial activity remaining inside the cells after 60 min), indicating a lower efficiency of ligand recycling in these cell lines. This, however, is most probably a consequence of the significantly lower hCXCR4 expression in HT-29 and MCF-7 cells compared to the transfected CHO cells, leading to less efficient tracer re-binding and reinternalization. Interestingly, a higher fraction of [^125^I]CPCR4.3 activity is ultimately retained in HT-29 and MCF-7 cells compared to the transfected cell lines under conditions inhibiting ligand recycling (25.1 ± 0.6% (HT-29) and 22.6 ± 1.0% (MCF-7) of the initial cellular activity after 120 min). The same effect had also been observed, when externalization kinetics of radioiodinated sst-ligands were investigated in sst_2_-transfected CHO cells and endogenously sst-expressing AR42J cells [22] and was attributed to differences in intracellular ligand trafficking between the cell lines.

Overall, [^125^I]CPCR4.3 shows suitable cellular uptake kinetics as well as retention both in hCXCR4 and in mCXCR4 expressing cells as a prerequisite for fast and specific h/mCXCR4 targeting in vivo as well as reasonably slow washout from target tissues.

### In vivo biodistribution

To assess the potential of [^125^I]CPCR4.3 to specifically target mCXCR4 expression in vivo, biodistribution studies using different mouse strains (Black Six: immunocompetent vs CD-1 nude: immunodeficient) were performed. Table 3 summarizes the biodistribution data obtained in Black Six mice.

**Table 3:** Biodistribution of [^125^I]CPCR4.3 in Black Six normal mice at 60 min p.i. (groups of *n* = 4). To determine CXCR4 specificity of tracer uptake, 50 μg AMD3100 (2 mg/kg) were coinjected. Data are cited as %iD/g and are means ± SD

Organ	Tracer only	Coinjection of AMD3100
Blood	0.39 ± 0.05	0.32 ± 0.11
Heart	0.36 ± 0.10	0.34 ± 0.10
Liver	21.18 ± 2.92	23.74 ± 4.68
Stomach	4.63 ± 2.94	1.08 ± 0.59
Pancreas	1.14 ± 0.87	0.42 ± 0.21
Intestine	30.36 ± 2.09	35.67 ± 2.48
Kidney	4.07 ± 0.25	4.70 ± 1.11
Lung	1.65 ± 0.25	0.49 ± 0.07*
Spleen	8.03 ± 2.91	2.78 ± 1.72*
Adrenals	1.43 ± 0.37	0.33 ± 0.14*
Muscle	0.13 ± 0.11	0.07 ± 0.05

*p < 0.005

[^125^I]CPCR4.3 shows rapid clearance from the circulation and is, due to its pronounced lipophilicity (log *P* = 0.51), predominantly cleared via the hepatobiliary route, leading to very high accumulation in liver and intestines. The only other organs with notable tracer accumulation are stomach, kidney, and spleen. As demonstrated by coinjection of an excess of unlabeled competitor, i.e., AMD3100, the splenic uptake of [^125^I]CPCR4.3 is primarily CXCR4 mediated. The same applies to [^125^I]CPCR4.3 uptake in the other tissues with endogenous CXCR4 expression, lung and adrenals [23]. In contrast, the reduction in tracer uptake by AMD3100 coinjection observed for stomach and pancreas is not statistically significant (*P* = 0.05 and 0.1, respectively).

**Fig. 4:**
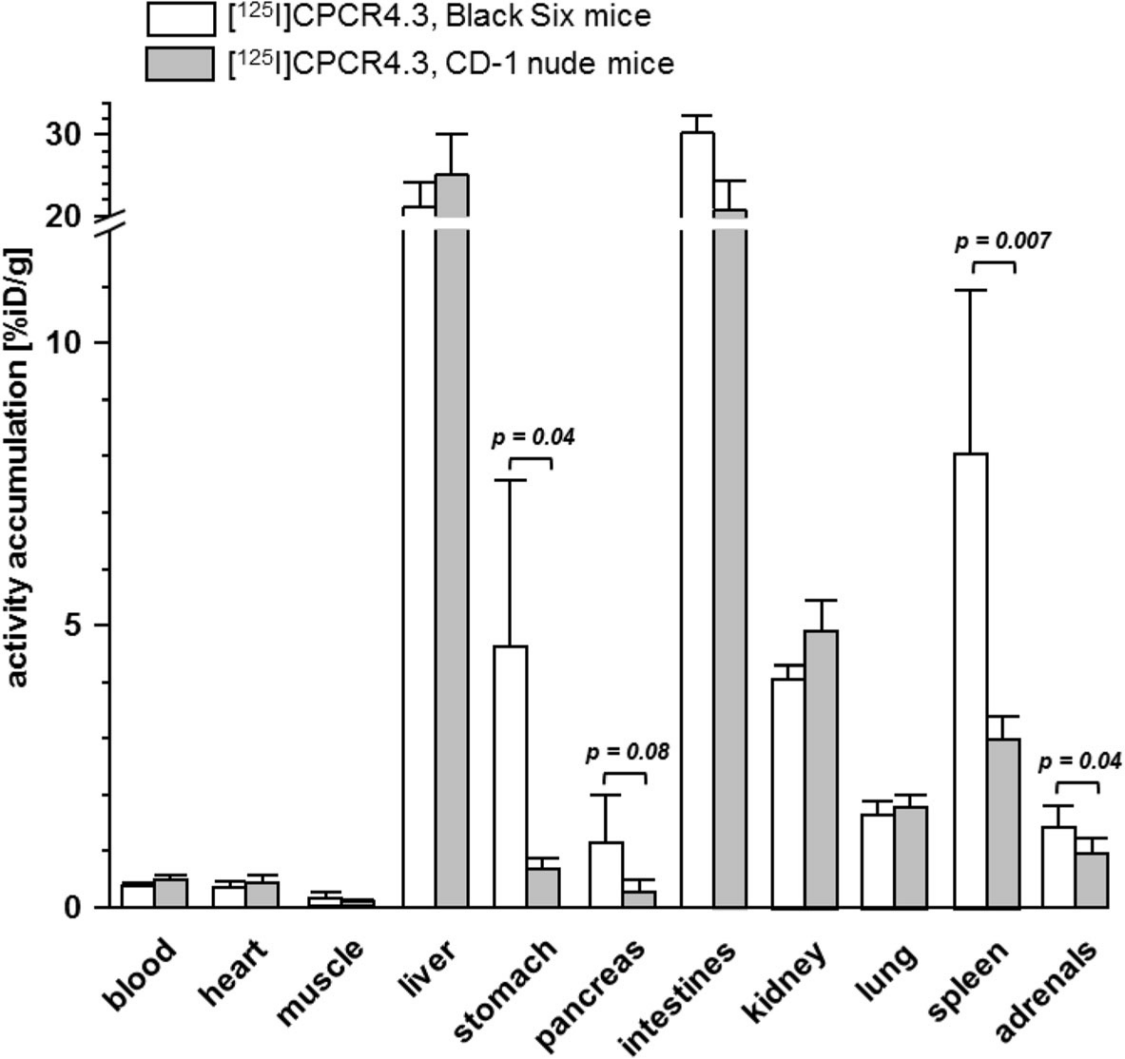
Biodistribution of [^125^I]CPCR4.3 in Black Six normal mice and CD-1 nude mice (*n* = 5) at 60 min p.i. (groups of *n* = 4). Data are given as %iD/g and are means ± SD

In the comparative biodistribution study in Black Six and CD-1 nude mice, no significant differences in the general biodistribution pattern of [^125^I]CPCR4.3 in non-target organs were observed (Fig. 4), except a slightly delayed hepatobiliary clearance of the tracer from liver to intestine in CD-1 mice. The only major and also highly relevant difference in the tissue accumulation of [^125^I]CPCR4.3 between mouse strains was observed in spleen; here, tracer accumulation in immunocompromised CD-1 mice was only 37% of the tracer uptake observed in Black Six mice. This finding is in line with the inability of immunocompromised CD1 nude mice to produce T cells, which account for app. 35% of the (mCXCR4 positive) immune cell population in mouse spleen. In contrast, [^125^I]CPCR4.3 uptake levels in the mCXCR4 expressing non-lymphoid organs lung and adrenals were not statistically different in the two mouse strains. This highlights the ability of [^125^I]CPCR4.3 to quantitatively detect—despite clearly not optimal excretion characteristics—different levels of endogenous mCXCR4 expression in vivo. This is also supported by results from a previous study, where [^125^I]CPCR4.3 was able to (semi) quantitatively detect murine immune cell infiltrates in a mouse model of esophageal carcinogenesis [24] via ex vivo autoradiography after intravenous tracer application.

## Conclusion

Due to its excellent hCXCR4 and mCXCR4 targeting efficiency, both in vitro and in vivo, [^125^I]CPCR4.3 represents a sensitive and reliable tool for the species-independent quantification of CXCR4 expression. Despite its suboptimal clearance properties due to its relatively high lipophilicity, i.e., high accumulation in the gastrointestinal system, which certainly restricts its use for in vivo imaging applications using ^123^I (for SPECT) or ^124^I (for PET), [^125^I]CPCR4.3 shows high and specific accumulation in mCXCR4 expressing tissues. Thus, it holds promise as a powerful preclinical tool for the investigation and quantification of CXCR4 involvement and kinetics in various murine disease models via, e.g., biodistribution and autoradiography studies. Given the successful proof-of-concept introduction of the CPCR4.3 pentapeptide scaffold as a high-affinity ligand for hCXCR4 and mCXCR4, ongoing efforts are currently directed toward the implementation of alternative radiolabeling strategies by refined chemical conjugation approaches, leading to first very promising radiometal-labeled CPCR4.3 analogs.
